# Supplementary material for: Phc2 controls hematopoietic stem and progenitor cell mobilization from bone marrow by repressing Vcam1 expression
Source: Nat Commun. 2019 Aug 2;10:3496. doi: 10.1038/s41467-019-11386-4 (PMC6677815; doi:10.1038/s41467-019-11386-4)
Supplement: Supplementary file 1 — Supplementary Information [file 41467_2019_11386_MOESM1_ESM.pdf]

Supplementary Information

Phc2 controls hematopoietic stem and progenitor cell mobilization from bone marrow by repressing *Vcam1* expression

Bae et al.

Supplementary Figure 1

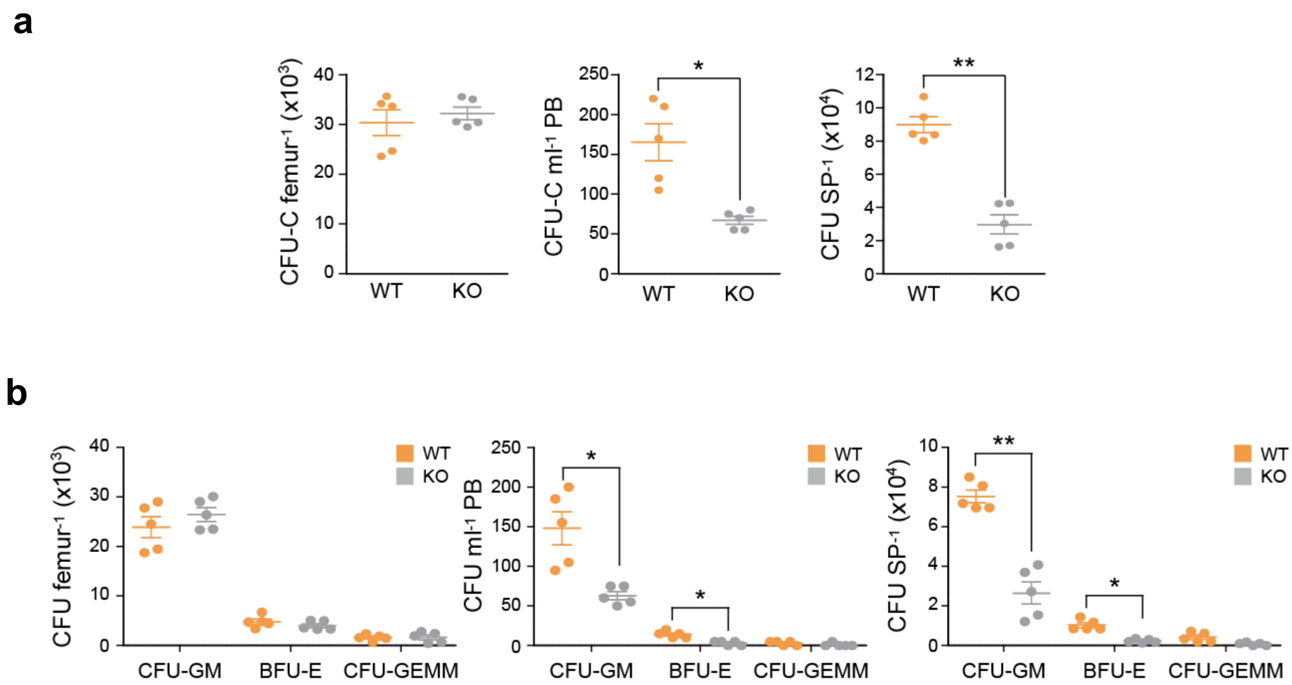

Supplementary Figure 1. CFU in the BM, PB, and spleen (SP) of WT and KO mice. **a** CFU-C. **b** CFU-GM, BFU-E and CFU-GEMM. $n = 5$. Statistical significance was assessed by two-tailed Student's t test. *$P < 0.05$; **$P < 0.01$. All data are presented as means ± SEM. Source data are provided as a Source Data File.

Supplementary Figure 2

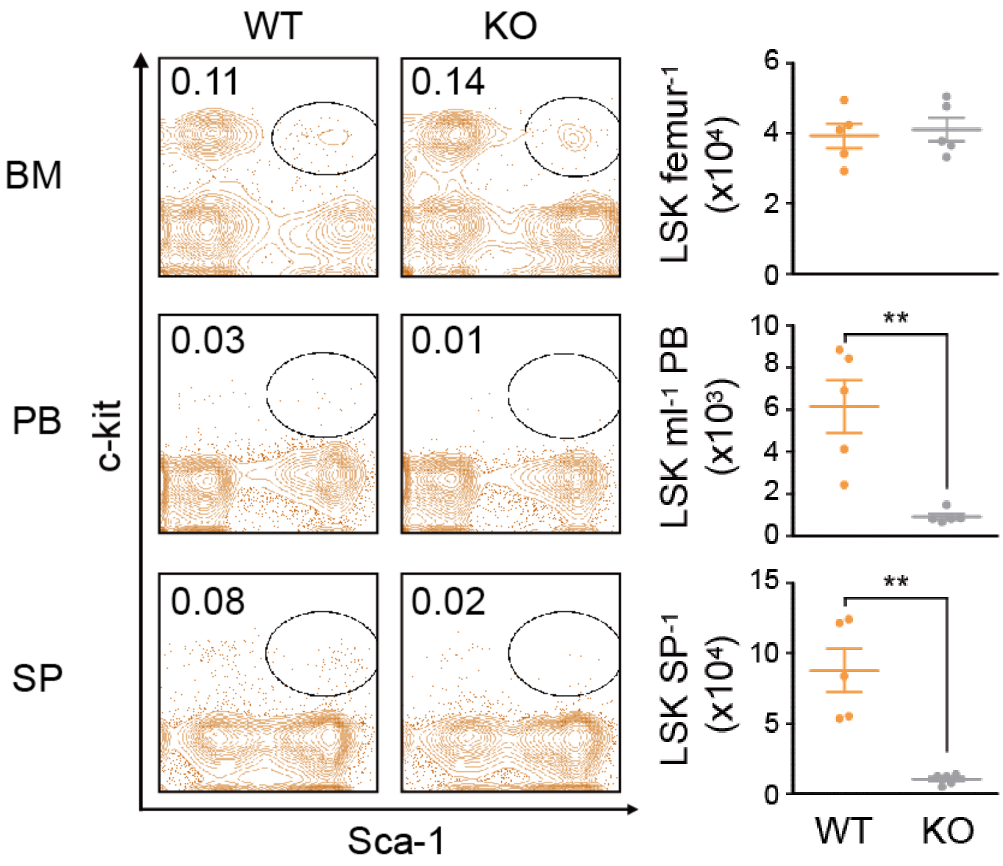

Supplementary Figure 2. Percentage (*left*) and absolute number (*right*) of LSK cells in the BM, PB, and spleen (SP) of WT and KO mice on day 5 after G-CSF treatment. $n = 5$. Representative image of flow cytometry analyses showing Sca-1 and c-kit staining of lineage negative cells. Percentages of LSK cells are indicated as numbers in each dot plot. Statistical significance was assessed by two-tailed Student's *t* test. $^{**}P < 0.01$. All data are presented as means ± SEM. Source data are provided as a Source Data File.

Supplementary Figure 3

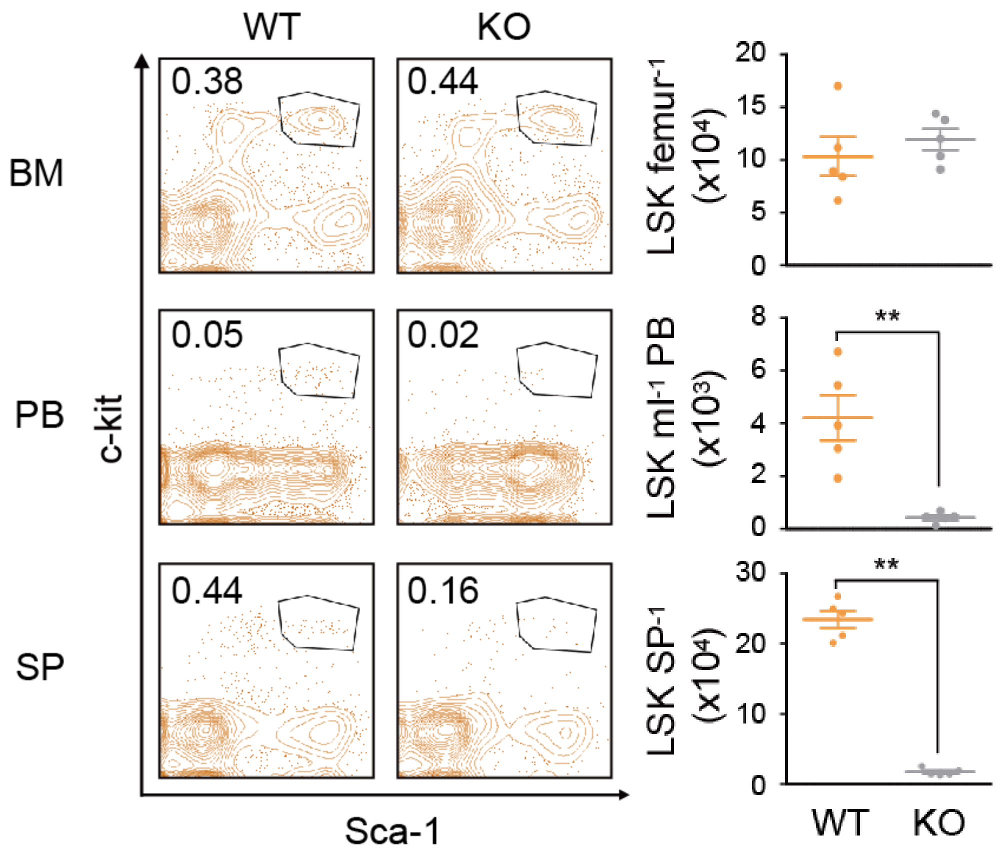

Supplementary Figure 3. Percentage (*left*) and absolute number (*right*) of LSK cells in the BM, PB, and spleen (SP) of WT and KO mice on day 16 after 5-FU treatment. $n = 5$. Representative image of flow cytometry analyses showing Sca-1 and c-kit staining of lineage negative cells. Percentages of LSK cells are indicated as numbers in each dot plot. Statistical significance was assessed by two-tailed Student's *t* test. $^{**}P < 0.01$. All data are presented as means ± SEM. Source data are provided as a Source Data File.

Supplementary Figure 4

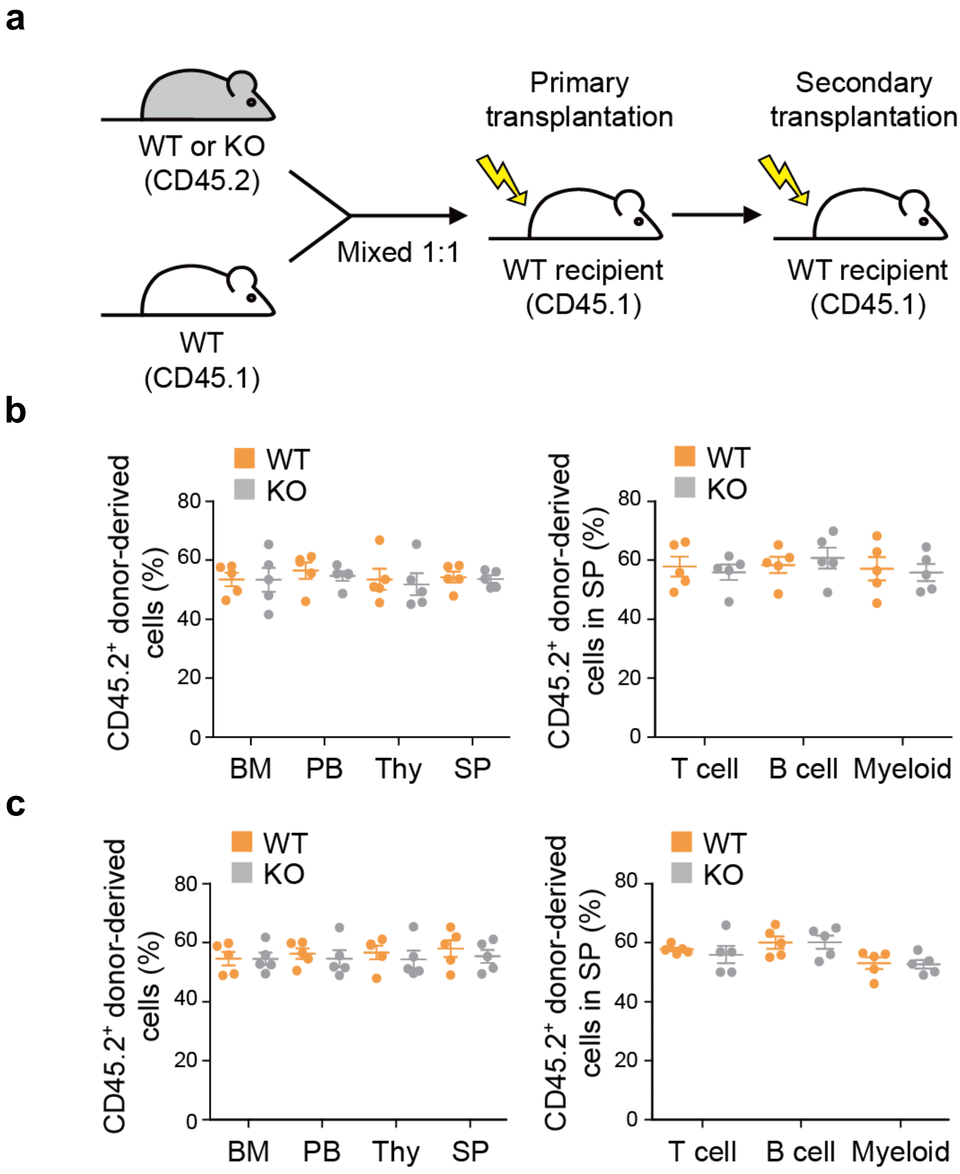

Supplementary Figure 4. No competitive disadvantage found in KO HSPCs. Serial competitive BM repopulation assays were performed using WT and KO mice. For primary transplantation, LSK cells (5×10^4) from WT and KO mice (CD45.2) were mixed with 5×10^4 competitor BM cells (CD45.1), and intravenously injected into lethally irradiated WT CD45.1 recipient mice. n = 5 per group. Secondary transplantation was performed at 12 weeks after primary engraftment. LSK cells (1×10^5) from primary transplants were intravenously injected into lethally irradiated WT CD45.1 recipient mice. n = 5 per group. **a** Schematic representation of serial competitive transplantation strategy. **b** Percentage of donor chimerism in BM, PB, thymus (Thy), and spleen (SP) of primary transplantation recipients (*left*) and percentage of donor-derived T cells, B cells, myeloid cells in SP of primary transplantation recipients (*right*). **c** Percentage of donor chimerism in BM, PB, Thy, and SP of secondary transplantation recipients (*left*) and percentage of donor-derived T cells, B cells, myeloid cells in SP of secondary transplantation recipients (*right*). Statistical significance was assessed by two-tailed Student's t test. All data are presented as means ± SEM. Source data are provided as a Source Data File.

Supplementary Figure 5

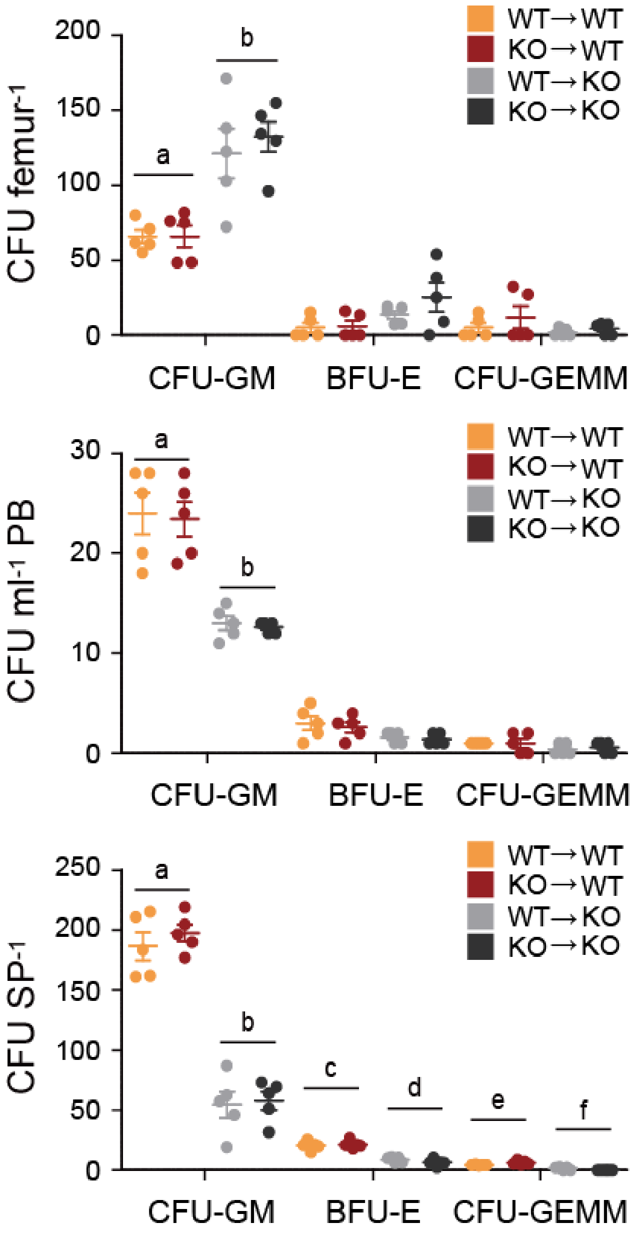

Supplementary Figure 5. The frequencies of donor-derived clonogenic progenitors homing to the BM (femur, *upper*), PB (*middle*) and SP (*lower*) of recipients at 16 h after CFSE-labeled LSK cell (WT or KO) injection into recipient mice (WT or KO). $n = 5$. Statistical significance was assessed by ANOVA analysis. Mean values not sharing the same superscript letter ($^{a\text{-}f}$) differ significantly at $P < 0.05$. All data presented as the means ± SEM. Source data are provided as a Source Data File.

Supplementary Figure 6

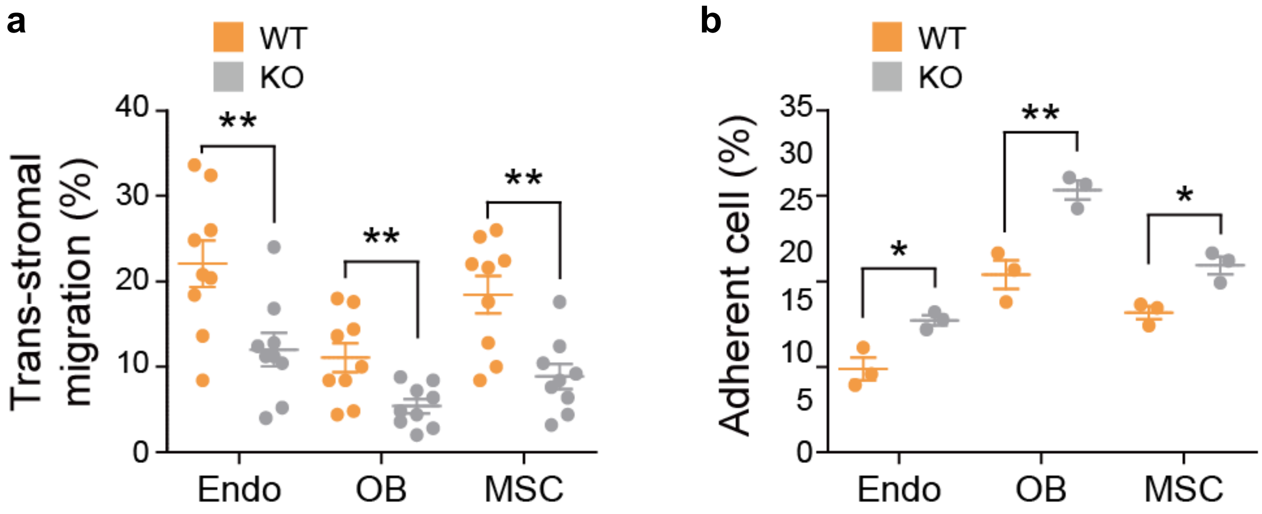

Supplementary Figure 6. Phc2 deficiency leads more firm adhesion between HSPCs and BMSCs in BM niches. Endothelial cells (Endo), osteoblasts (OB) or mesenchymal stem cells (MSC) in WT or KO BM were isolated by FACS sorting. Then, trans-stromal migration assays were performed to measure the capacity of WT LSK cells to transmigrate across each subpopulation of WT or KO BMSCs. **a** Relative ratio of migrated WT LSK cells through each subpopulation of WT or KO BMSCs. $n = 9$. **b** Relative ratio of adhered WT LSK cells to each subpopulation of WT or KO BMSCs. $n = 3$. Statistical significance was assessed by two-tailed Student's *t* test. *$P < 0.05$; **$P < 0.01$. All data are presented as means ± SEM. Source data are provided as a Source Data File.

Supplementary Figure 7

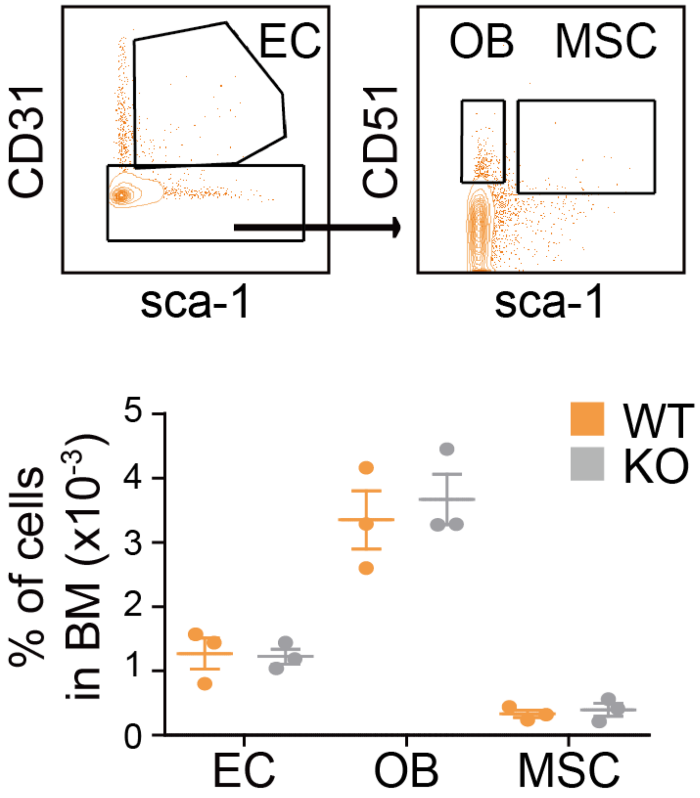

Supplementary Figure 7. The composition of BMSCs is not changed in BM niches of KO mice. Representative image of flow cytometry analysis showing Sca-1, CD31, and CD51 staining of lineage negative and CD45 negative cells to identify BMSCs (*upper panel*). Frequencies of endothelial cells (EC), osteoblasts (OB) and mesenchymal stem cells (MSC) in BM of WT and KO mice are shown in the *lower* panel. $n = 3$. Statistical significance was assessed by two-tailed Student's *t* test. All data are presented as means ± SEM. Source data are provided as a Source Data File.

Supplementary Figure 8

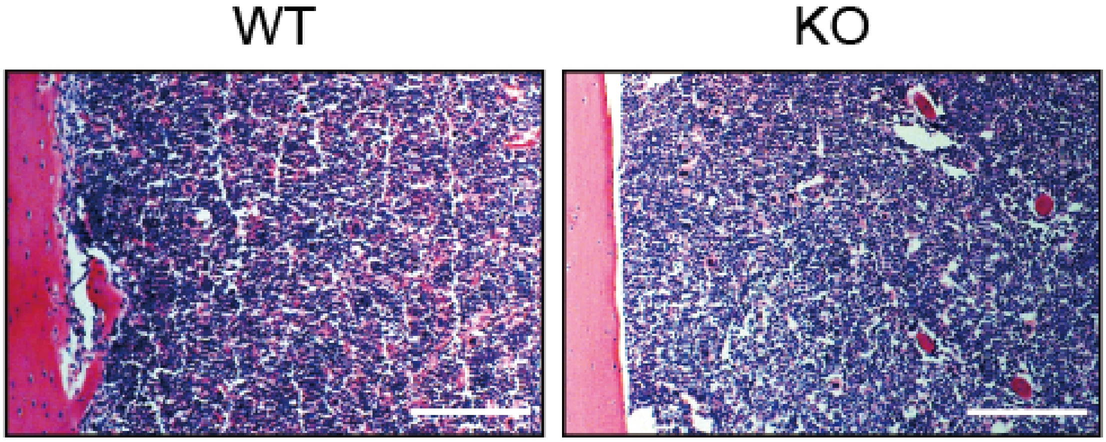

Supplementary Figure 8. Normal architecture of BM of KO mice. Representative hematoxylin and eosin staining images of BM sections from WT and KO mice n = 5. *Scale bar:* 200 µm.

Supplementary Figure 9

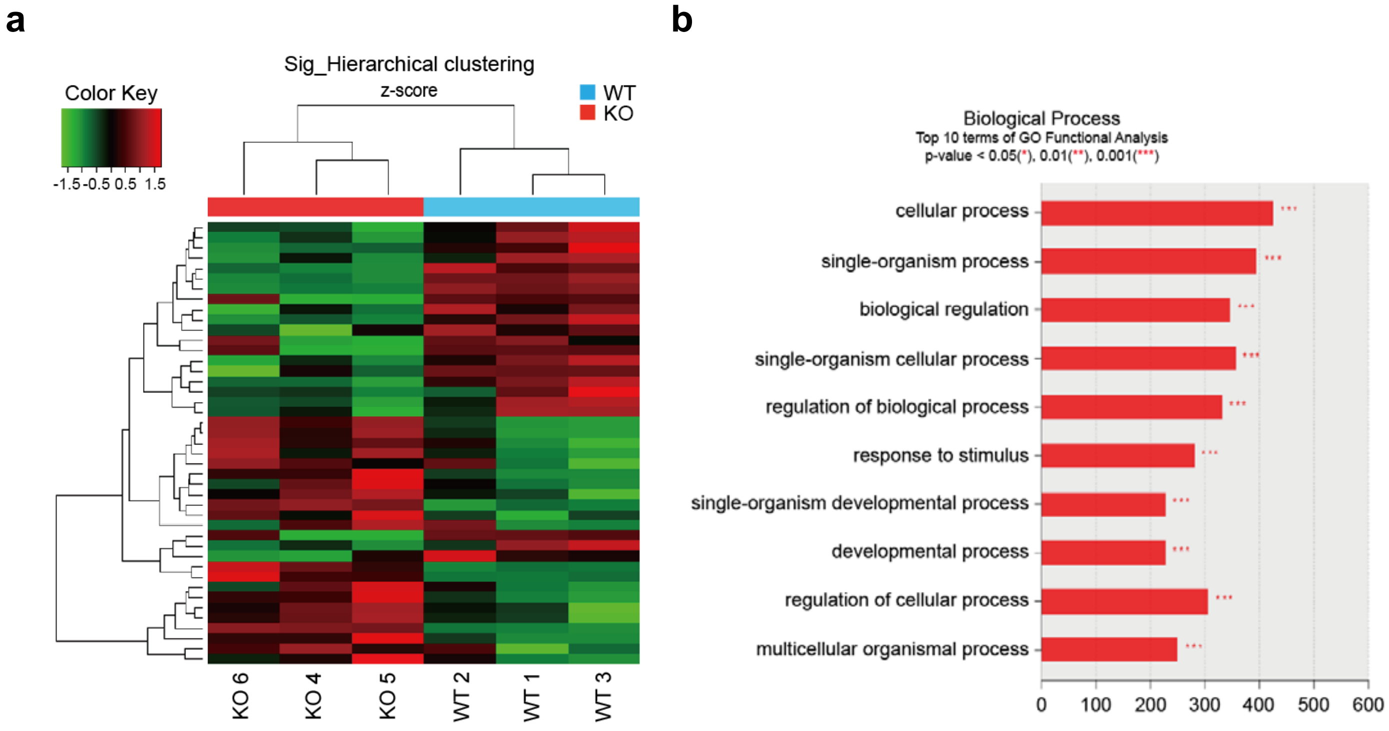

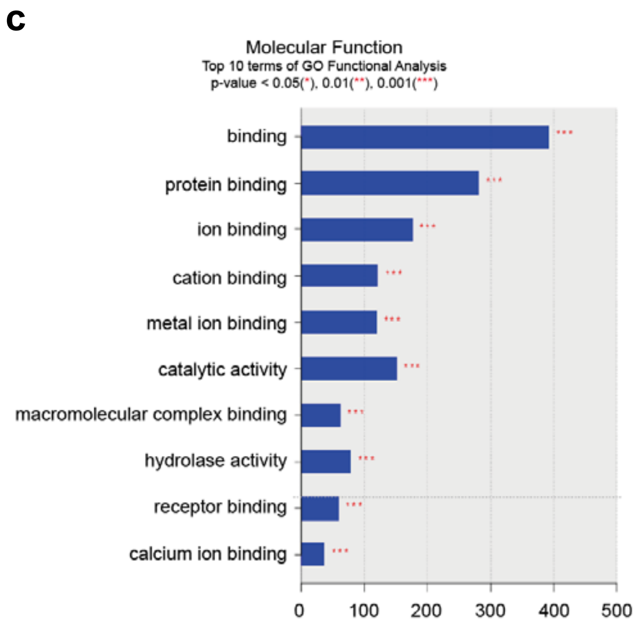

d

Gene symbol	Description	fold change	*p*-value
Alcam	activated leukocyte cell adhesion molecule	1.02	0.8007
Angpt1	angiopoietin 1	-1.17	0.1201
Cdh2	cadherin 2	-1.22	0.7244
Ctsk	cathepsin K	-1.02	0.9721
Cxcl12	chemokine (C-X-C motif) ligand 12	-1.16	0.5294
Fn1	fibronectin 1	1.11	0.6834
Kitl	kit ligand	-1.08	0.8041
Icam1	intercellular adhesion molecule 1	-1.04	0.7501
Selp	selectin, platelet	-1.12	0.6364
Vcam1	vascular cell adhesion molecule 1	1.46	0.0028

Supplementary Figure 9. Overview of RNA seq and differential expression analysis in WT and KO BM cells. **a** Hierarchical clustering analysis of differentially expressed genes (DEGs) between WT and KO BM cells. 'Red' indicates relatively higher expression and 'green' indicates relatively lower expression. **b, c** GO functional enrichment analysis according to biological process (**b**) and molecular function (**c**). X axis represents DEG count while Y axis represents GO terms. **d** mRNA expression patterns of genes related to HSPC mobilization in WT and KO BM cells. '+' value means increased mRNA expression of indicated gene in KO BM cells compared to that in WT BM cells, whereas '-' value means decreased mRNA expression of indicated gene in KO BM cells compared to that in WT BM cells.

Supplementary Figure 10

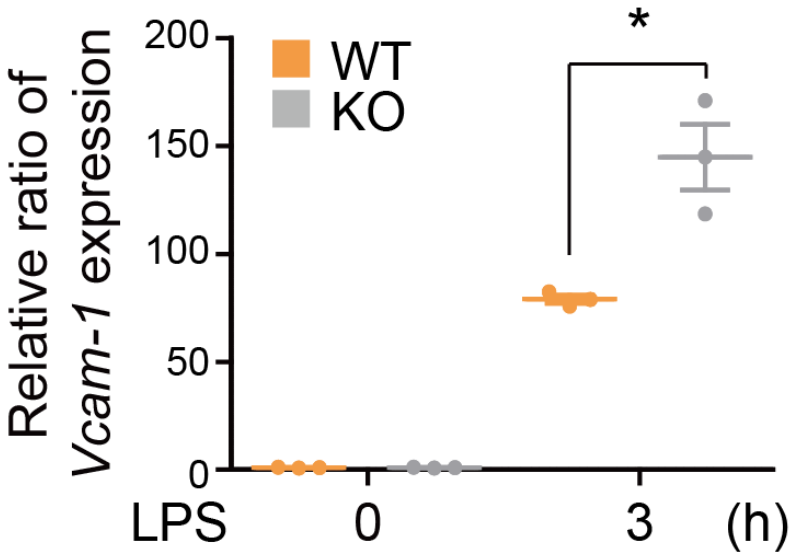

Supplementary Figure 10. Relative fold change of *Vcam1* mRNA expression in LPS-stimulated peritoneal macrophages from WT or KO mice. WT or KO peritoneal macrophages were harvested on day 3 after intraperitoneal injection of thioglycolate (3%). Isolated macrophages (2 x 10^6 cells $well^{-1}$) were seeded and stimulated with LPS (1 µg ml^{-1}) in 6 well plate. Three h after stimulation, total RNA of each sample was isolated to monitor *Vcam1* mRNA expression by real-time qRT-PCR analysis. $n = 3$. The level of *Vcam1* mRNA transcripts was normalized with that of *Gapdh.* Relative fold change of each *Vcam1* mRNA expression from WT or KO macrophages was calculated relative to basal level of that in unstimulated WT or KO macrophages, respectively. Statistical significance was assessed by two-tailed Student's *t* test. $^*P < 0.05$. All data are presented as means ± SEM. Source data are provided as a Source Data File.

Supplementary Figure 11

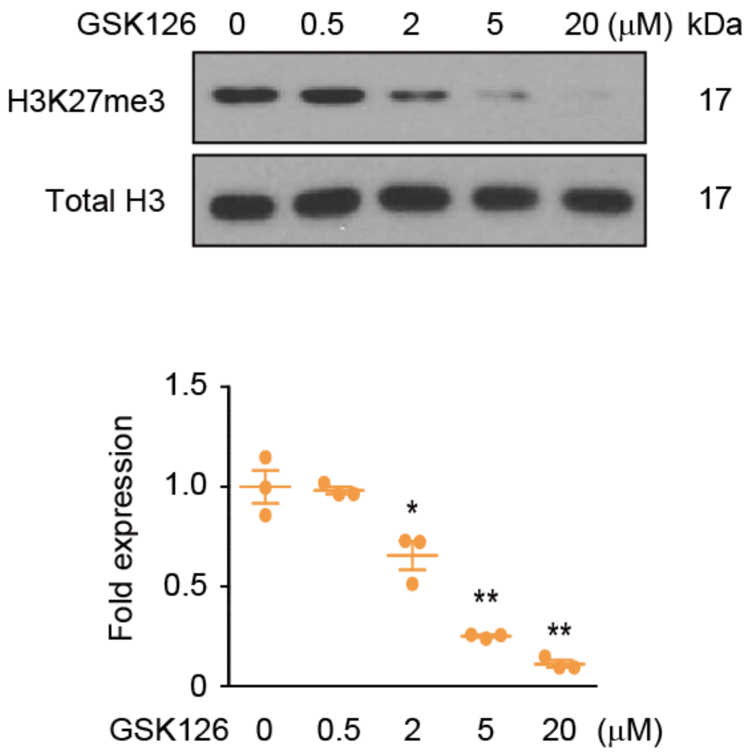

Supplementary Figure 11. GSK126 inhibits cellular H3K27me3 in a dose dependent manner in BMSCs. BMSCs (OP9 cells) were treated with various concentrations of GSK126 for 48 h. After treatment, cell lysates were subjected to immunoblot analyses using anti-H3K27me3 and anti-histone H3 antibody (*upper*). To quantify inhibitory effect of GSK126 on H3K27me3, immunoreactive bands were normalized to total H3 levels (*lower*). $n = 3$. Statistical significance was assessed by two-tailed Student's *t* test. *$P < 0.05$; **$P < 0.01$. All data are presented as means ± SEM. Source data are provided as a Source Data File.

Supplementary Figure 12

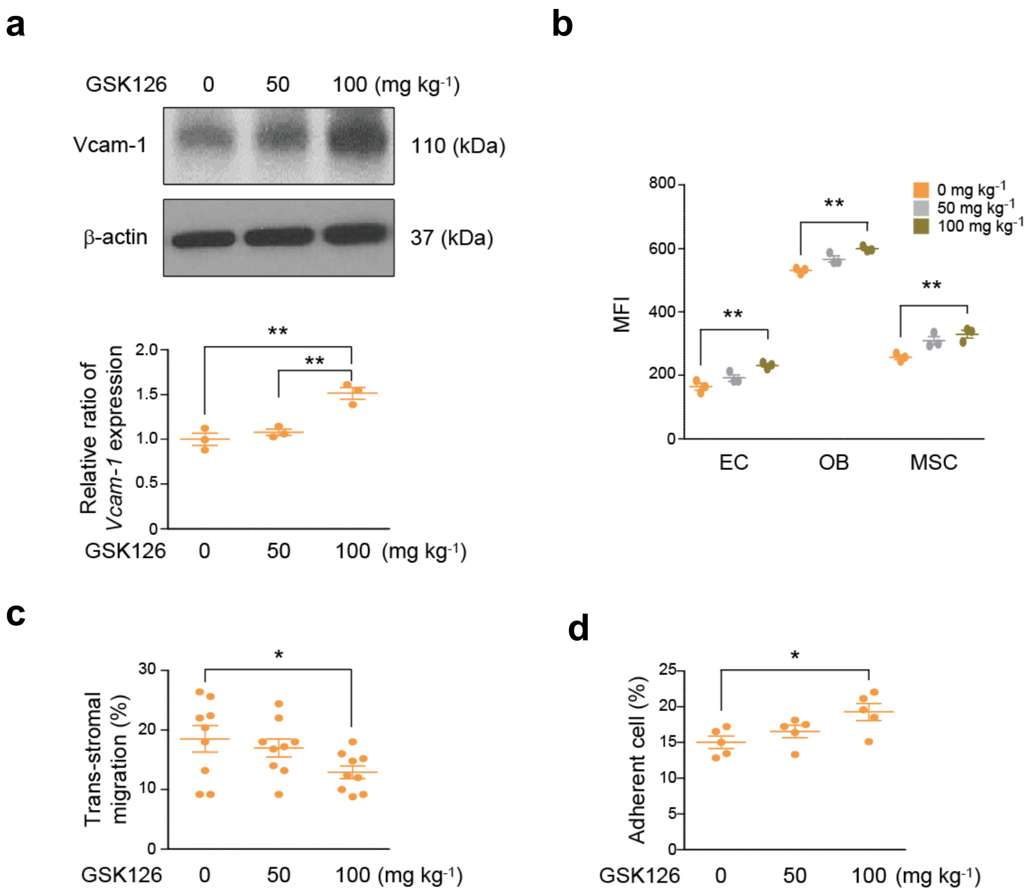

Supplementary Figure 12. *In vivo* administration of GSK126 into WT mice mimics the phenotype of KO mice. WT mice were scarified on 16 h after intraperitoneal injection of GSK126 (50 mg kg^{-1} or 100 mg kg^{-1}) to monitor VCAM-1 expression in BMSCs and perform trans-stromal migration and adhesion assays. **a, b** Relative ratio of VCAM-1 expression in BMSCs isolated from GSK126 injected mice was analyzed by immunoblot (**a**) and flow cytometry (**b**). $n = 3$. EC, endothelial cells; OB, osteoblasts; MSC, mesenchymal stem cells. **c** Relative ratio of migrated LSK cells through BMSCs isolated from GSK126 injected mice. $n = 9$. **d** Relative ratio of adhered LSK to BMSCs isolated from GSK126 injected mice. $n = 5$. Statistical significance was assessed by two-tailed Student's *t* test. *$P < 0.05$; **$P < 0.01$. All data are presented as means ± SEM. Source data are provided as a Source Data File.

Supplementary Figure 13

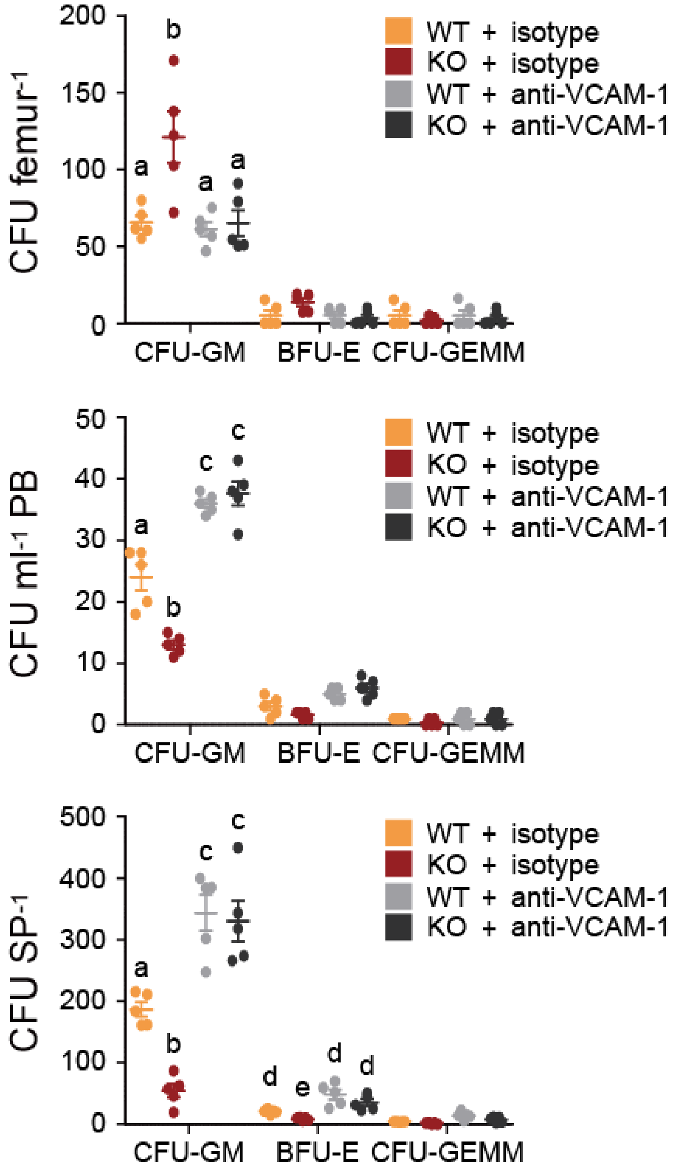

Supplementary Figure 13. *In vivo* homing assay using mice administered with anti-VCAM-1 antibody. CFSE-labeled WT LSK cells were intravenously injected into lethally irradiated WT or KO recipient mice. Anti-VCAM-1 Ab or its isotype control Ab (2 mg kg^{-1}) was intravenously injected into recipient mice 1 h before adoptive cell transfer. Sixteen h after adoptive cell transfer, CFU assays were performed to measure the frequencies of donor-derived clonogenic progenitors homing to the BM (femur, *upper*), PB (*middle*) and SP (*lower*) of recipients. $n = 5$. WT, WT recipient mice; KO, KO recipient mice. Isotype, isotype antibody treatment; anti-VCAM-1, anti-VCAM-1 antibody treatment. Statistical significance was assessed by ANOVA analysis. Mean values not sharing the same superscript letter ($^{a-e}$) differ significantly at $p < 0.05$. All data presented as the means ± SEM. Source data are provided as a Source Data File.

Supplementary Figure 14

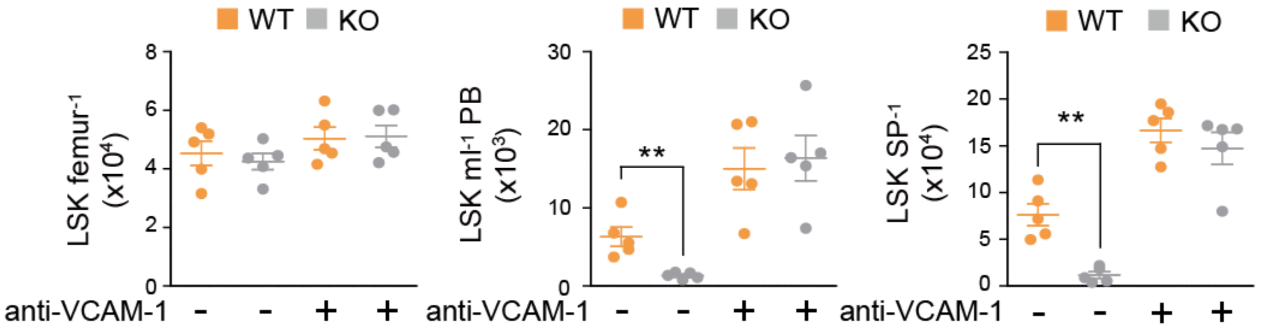

Supplementary Figure 14. Absolute number of LSK cells in the BM, PB, and spleen (SP) of WT and KO mice on day 5 after G-CSF treatment. Anti-VCAM-1 Ab or an isotype control Ab (2 mg kg^{-1} day^{-1}) was daily administered intravenously into WT and KO recipient mice. $n = 5$. "-", treatment with an isotype control Ab; "+", treatment with anti-VCAM-1 Ab. Statistical significance was assessed by two-tailed Student's *t* test. $^{**}P < 0.01$. All data are presented as means ± SEM. Source data are provided as a Source Data File.

Supplementary Figure 15

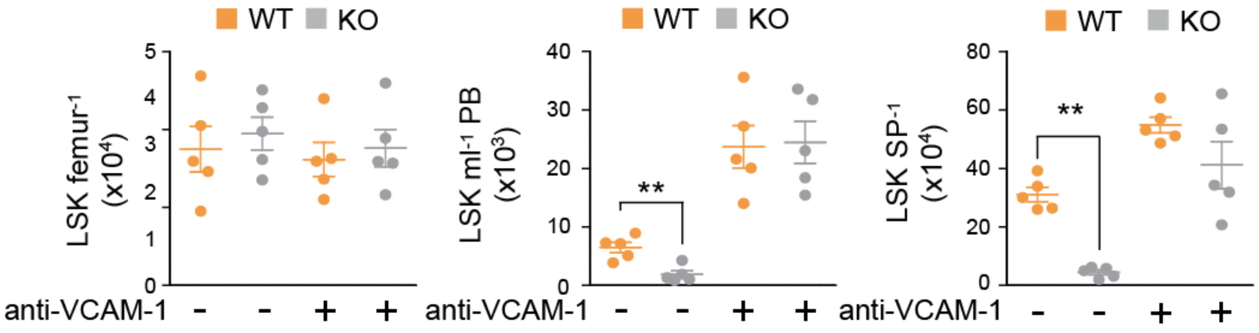

Supplementary Figure 15. Absolute number of LSK cells in the BM, PB, and spleen (SP) of WT and KO mice on day 16 after 5-FU treatment. Anti-VCAM-1 Ab or an isotype control Ab (2 mg $kg^{-1}day^{-1}$) was daily administered intravenously into WT and KO recipient mice. $n = 5$. "-", treatment with an isotype control Ab; "+", treatment with anti-VCAM-1 Ab. Statistical significance was assessed by two-tailed Student's t test. $**P < 0.01$. All data are presented as means ± SEM. Source data are provided as a Source Data File.

Supplementary Figure 16

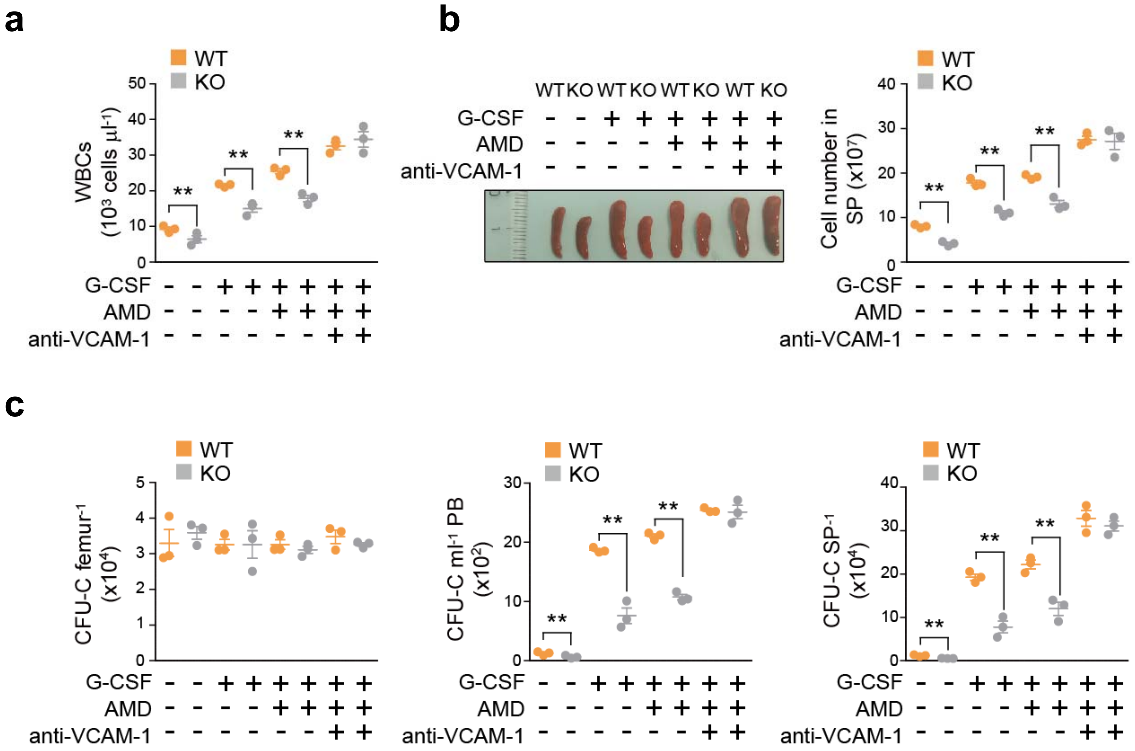

Supplementary Figure 16. Administration of anti-VCAM-1 Ab reverses the defect involving G-CSF and AMD3100-induced HSPC mobilization in KO mice. G-CSF and AMD3100-induced HSPC mobilization assay with anti-VCAM-1 Ab is described in "*Methods*" section. $n = 3$ per group. **a** WBC counts from experimental mice administered with G-CSF, AMD3100 and/or anti-VCAM-1 Ab. **b** Representative image of spleens (*left*) and absolute number of splenocytes (*right*) from experimental mice administered with G-CSF, AMD3100 and/or anti-VCAM-1 Ab. **c** CFU-C in the BM (femur, *left*), PB (*middle*), and spleen (SP, *right*) from experimental mice administered with G-CSF, AMD3100 and/or Ab. "-", treatment with vehicle or isotype control Ab; "+", treatment with G-CSF or anti-VCAM-1 Ab. Statistical significance was assessed by two-tailed Student's *t* test. $^{**}P < 0.01$. All data are presented as means ± SEM. Source data are provided as a Source Data File.

Supplementary Figure 17

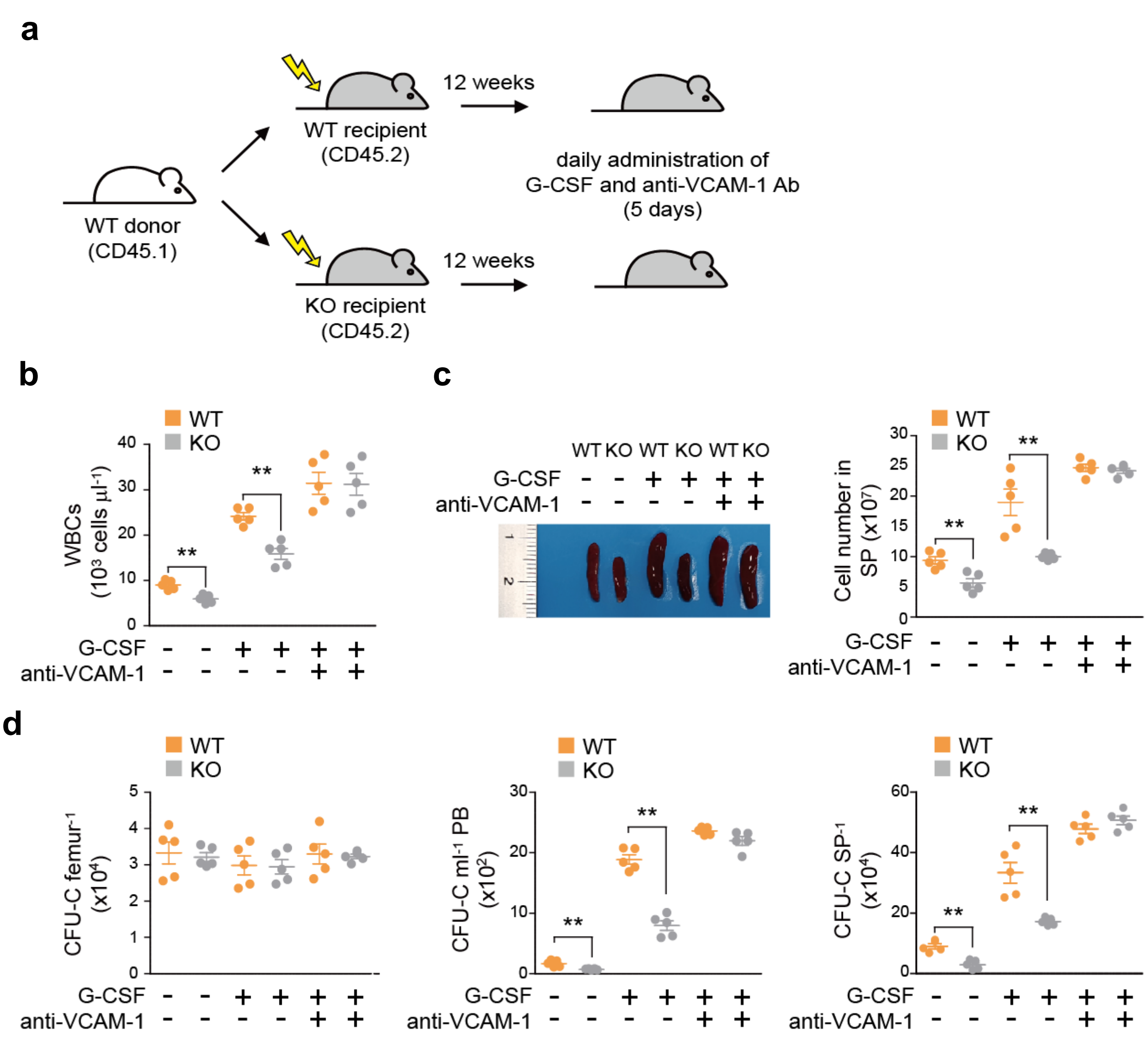

Supplementary Figure 17. G-CSF induced HSPC mobilization assay in BM chimeric mice. LSK cells (1×10^5) from WT mice (CD45.1) were injected intravenously into lethally irradiated WT or KO CD45.2 recipient mice. Twelve weeks later, G-CSF or vehicle was administered by daily subcutaneous injection to chimeric mice at a dose of 250 µg kg^{-1} day^{-1} for 5 days. To block the VLA-4 and VCAM-1 interaction, anti-VCAM-1Ab or the respective isotype control (2 mg kg^{-1} day^{-1}) was administered intravenously every day. $n = 5$ per group. **a** Schematic representation of LSK transplantation and G-CSF induced mobilization assay. **b** Comparison of WBC counts between BM chimeric mice on day 5 after G-CSF and/or anti-VCAM1 Ab treatment. **c** Representative image of spleens from BM chimeric mice (*left*) and absolute numbers of splenocytes for BM chimeric mice (*right*) on day 5 after G-CSF and/or anti-VCAM1 Ab treatment. **d** CFU-C in the BM (femur, *left*), PB (*middle*), and spleen (SP, *right*) of BM chimeric mice on day 5 after G-CSF and anti-VCAM1 Ab treatment. WT, WT recipient mice; KO, KO recipient mice. "-", treatment with vehicle or isotype control Ab; "+", treatment with G-CSF or anti-VCAM-1 Ab. Statistical significance was assessed by two-tailed Student's *t* test. $^{**}P < 0.01$. All data are presented as means ± SEM. Source data are provided as a Source Data File.

Supplementary Figure 18

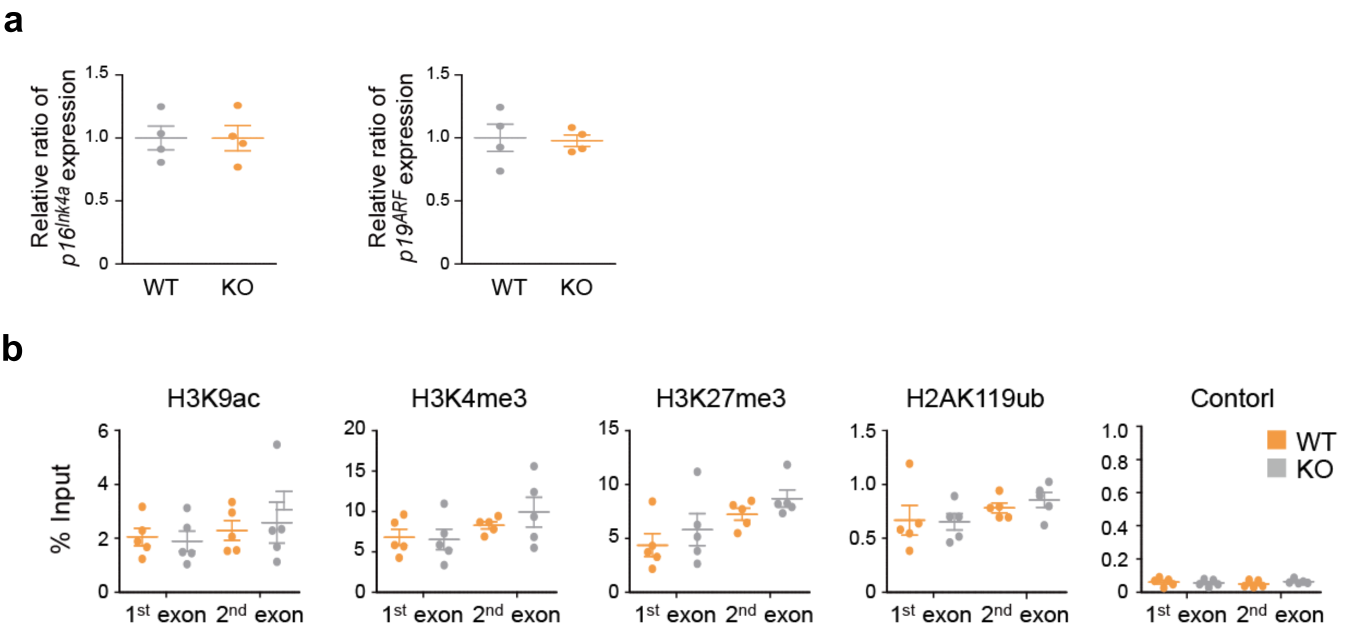

Supplementary Figure 18. The expression and epigenetic pattern of *Cdkn2a* in KO BM cells show no significant differences compared to those in WT BM cells. **a** mRNA expression patterns of $p16^{Ink4a}$ (*left*) and $p19^{ARF}$(*right*) in WT or KO BM cells were accessed by qRT-PCR. $n = 4$. **b** ChIP was performed with WT or KO BM cell lysates using indicated Abs or a control Ab (Control). DNA recovered from ChIP was then analyzed by qPCR. $n = 5$. 1st exon, $P16^{Ink4a}$ first exon; 2nd exon, $P16^{Ink4a}/p19^{ARF}$ common second exon. The primer pairs used for qRT-PCR are listed in Supplementary Table 1. Statistical significance was assessed by two-tailed Student's *t* test. All data are presented as means ± SEM. Source data are provided as a Source Data File.

Supplementary Figure 19

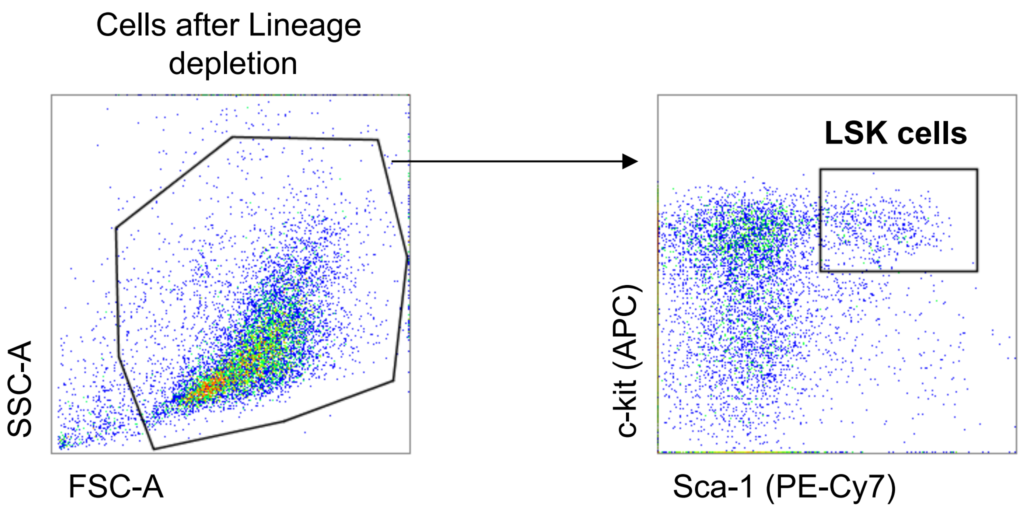

Supplementary Figure 19. Gating strategy for LSK cell sorting. Lineage positive cells in BM were depleted using a lineage-specific Ab cocktail coupled with anti-biotin microbeads by MACS cell separation system. After depletion, lineage negative cells were stained with Sca-1 and c-kit antibodies. Double positive (Sca-1^{+}c-kit^{+}) cells were then isolated using FACSAria Fusion Cell Sorter.

Table S1 Primers for qPCR analyses

Gene	Forward Primer	Reverse Primer
Alcam	5′-CTGATTGTGGGAATTGTCGTT-3′	5'-TCTTAGGCTTCTGTTTTGTCATTGT-3'
Angpt1	5'-GGAAGATGGAAGCCTGGAT-3'	5'-CCTTCCCAGTCCATCAGC-3'
Cdh2	5′-CCTGAGATACAGCGTCACTGG-3′	5′-GCTATCAGCTCTCGATCCAG-3′
Ctsk	5'-CGAAAAGAGCCTAGCGAACA-3'	5'-TGGGTAGCAGCAGAAACTTG-3'
Cxcl12	5'-GGTTCTTCGAGAGCCACATC-3'	5'-TGGGCTGTTGTGCTTACTTG-3'
Fn1	5′-ACGGACATCTGTGGTGTAGCA-3′	5′-GCACAGAGCACCATTGGAA-3′
Kitl	5'-GCTGCTGGTGCAATATGCT-3'	5'-TCCTTGGTTTTGACAAGAGGAT-3'
Icam1	5'-AGCCTCCGGACTTTCGAT-3'	5'-GAGCTTCAGAGGCAGGAAAC-3'
Phc2	5′-GCCTACAAGTTCAAGCGTTCC-3′	5′-TCTGCAGCTTGCTTCGGTC-3′
Selp	5'-CCTGGCAAGTGGAATGATGA-3'	5'-GAGCAGGTATAGCTCCCAA-3'
Vcam1	5'-TGGTGAAATGGAATCTGAACC-3'	5'-GACCCAGATGGTGGTTTCC-3'
Gapdh	5′-ATGGTGAAGGTCGGTGTGAA-3′	5′-GGTCGTTGATGGCAACAATCTC-3′

Table S2 Primers for ChIP assays

Gene	Forward Primer	Reverse Primer
Vcam1 #1	5'-CTCTCACGTGGCCTTATGAAC-3'	5'-CCTGCAGCATCTTCTAGATG-3'
Vcam1 #2	5'-GACTTCCTGTCATCCAGCAA-3'	5'-GGACGTTGATGCAGAATGAAGG-3'
Vcam1 #3	5'-GTGATCTCTGTCTTTGCCTGTC-3'	5'-TTCCTGACCTTCAGCTCCGAA-3'
Vcam1 #4	5'-GATTCCAGCACCTTCCTCAAGA-3'	5'-TGCGCAGCAATACAGTGTCC-3'
Vcam1 #5	5'-AATGGCTCTTCCTATGCTCTGC-3'	5'-TGGAACAGGTCATTGTCACAGC-3'
Vcam1 #6	5'-CCACCATTGAAGATACCGGGAA-3'	5'-GCTGTAGAGTCTGCCTCTGTT-3'
Vcam1 #7	5'-ATGAGTATTGCAACCACGAGCTG-3'	5'-AAGCTGCACTGAGGACCATCG-3'
