# Supplementary material for: Phc2 controls hematopoietic stem and progenitor cell mobilization from bone marrow by repressing Vcam1 expression
Source: Nat Commun. 2019 Aug 2;10:3496. doi: 10.1038/s41467-019-11386-4 (PMC6677815; doi:10.1038/s41467-019-11386-4)
Supplement: Supplementary file 3 — Reporting Summary [file 41467_2019_11386_MOESM3_ESM.pdf]

nature research

Corresponding author(s): DBPR NCOMMS-18-11826A

Last updated by author(s): Jun 11, 2019

Reporting Summary

Nature Research wishes to improve the reproducibility of the work that we publish. This form provides structure for consistency and transparency in reporting. For further information on Nature Research policies, see Authors & Referees and the Editorial Policy Checklist.

Statistics

For all statistical analyses, confirm that the following items are present in the figure legend, table legend, main text, or Methods section.

n/a	Confirmed	
☐	☒	The exact sample size (*n*) for each experimental group/condition, given as a discrete number and unit of measurement
☐	☒	A statement on whether measurements were taken from distinct samples or whether the same sample was measured repeatedly
☐	☒	The statistical test(s) used AND whether they are one- or two-sided<br>*Only common tests should be described solely by name; describe more complex techniques in the Methods section.*
☒	☐	A description of all covariates tested
☐	☒	A description of any assumptions or corrections, such as tests of normality and adjustment for multiple comparisons
☐	☒	A full description of the statistical parameters including central tendency (e.g. means) or other basic estimates (e.g. regression coefficient) AND variation (e.g. standard deviation) or associated estimates of uncertainty (e.g. confidence intervals)
☐	☒	For null hypothesis testing, the test statistic (e.g. F, t, r) with confidence intervals, effect sizes, degrees of freedom and P value noted<br>*Give P values as exact values whenever suitable.*
☒	☐	For Bayesian analysis, information on the choice of priors and Markov chain Monte Carlo settings
☒	☐	For hierarchical and complex designs, identification of the appropriate level for tests and full reporting of outcomes
☒	☐	Estimates of effect sizes (e.g. Cohen's *d*, Pearson's *r*), indicating how they were calculated

Our web collection on statistics for biologists contains articles on many of the points above.

Software and code

Policy information about availability of computer code

Data collection	Collection of flow cytometry data: CellQuest software
Data analysis	Flow cytometry : FlowJo (version 10.07)<br>WB image analysis: Image J (version 1.32j)<br>Data visualization : GraphPad Prism (version 5.03)<br>RNA seq raw reads alignment: HISAT (version 2.0.5)<br>Assemble transcripts and estimate FPKM: StringTie (version 1.3.3b)<br>statistical analysis : SPSS statistics softwere (version 24.0)

For manuscripts utilizing custom algorithms or software that are central to the research but not yet described in published literature, software must be made available to editors/reviewers. We strongly encourage code deposition in a community repository (e.g. GitHub). See the Nature Research guidelines for submitting code & software for further information.

Data

Policy information about availability of data

All manuscripts must include a data availability statement. This statement should provide the following information, where applicable:

- Accession codes, unique identifiers, or web links for publicly available datasets
- A list of figures that have associated raw data
- A description of any restrictions on data availability

The raw mRNA-seq data were deposited into the Sequence Read Archive (SRA) of National Center for Biotechnology Information (NCBI) with the following accession numbers: GSM3683312, GSM3683313, GSM3683314, GSM3683315, GSM3683316 and GSM3683317. An overview of the gene expression data was deposited at NCBI's Gene Expression Omnibus (GEO). It is accessible through GEO series accession number of GSE128705 (https://www.ncbi.nlm.nih.gov/geo/query/acc.cgi?acc=GSE128705). Source data underlying figures 1-8, tables 1-2, and supplementary figures 1-18 are provided as a Source Data file. All other data that support the findings of this study are available from the corresponding author upon reasonable request.

Field-specific reporting

Please select the one below that is the best fit for your research. If you are not sure, read the appropriate sections before making your selection.

☒ Life sciences ☐ Behavioural & social sciences ☐ Ecological, evolutionary & environmental sciences

For a reference copy of the document with all sections, see nature.com/documents/nr-reporting-summary-flat.pdf

Life sciences study design

All studies must disclose on these points even when the disclosure is negative.

Sample size	No statistical analysis methods were used to predetermine sample size estimates. Sample size was determined to be adequate based on the magnitude and consistency of measurable differences between groups.
Data exclusions	No data excluded
Replication	Each experiment was repeated at least three times as described in Figure legends.
Randomization	Mice were assigned according to their genotype. Litter mates and sex and age-matched animals were used whenever possible. All other parameters are random.
Blinding	N/A

Reporting for specific materials, systems and methods

We require information from authors about some types of materials, experimental systems and methods used in many studies. Here, indicate whether each material, system or method listed is relevant to your study. If you are not sure if a list item applies to your research, read the appropriate section before selecting a response.

Materials & experimental systems

n/a	Involved in the study	
☐	☒	Antibodies
☐	☒	Eukaryotic cell lines
☒	☐	Palaeontology
☐	☒	Animals and other organisms
☒	☐	Human research participants
☒	☐	Clinical data

Methods

n/a	Involved in the study	
☒	☐	ChIP-seq
☐	☒	Flow cytometry
☒	☐	MRI-based neuroimaging

Antibodies

Antibodies used	anti-CD16/32 (Purified, clone 2.4G2, BD Biosciences 553141), anti-B220 (PE, clone RA3-6B2, BD Biosciences 553090), anti-CD3ε (FITC, clone 146-2C11, Thermo Fisher Scientific 11-0031-63), anti-CD4 (FITC, clone GK1.5, BD Bioscience 553729), anti-CD8 (FITC, clone 53-6.7, BD Biosciences 553030), anti-CD11b (FITC, clone M1/70, Thermo Fisher Scientific 11-0112-41), anti-CD11c (PE, clone HL3, BD Biosciences 553802), anti-CD16/32 (Purified, clone 93, Thermo Fisher Scientific 14-0161-81), anti-CD25 (APC, clone PC61.5, Thermo Fisher Scientific 17-0251-81), anti-CD31 (APC, clone MEC 13.3, BD Biosciences 561814), anti-CD34 (FITC, clone RAM34BD Biosciences 560238), anti-CD44 (PE-Cy™5, clone IM7, BD Biosciences 561861), anti-CD45 (Purified, clone 30-F11, BD Biosciences 550539), anti-CD48 (APC, clone HM48-1, Biolegend 103412), anti-CD51 (PE, clone RMV-7, BD Biosciences 551187), anti-CD106 (FITC, clone 429, BD Biosciences 553332), anti-CD127 (PerCP-Cy5.5, clone A7R34, Thermo Fisher Scientific 45-1271-80), anti-CD150 (PerCP-Cy5.5, clone TC15-12F12.2, Biolegend 115922), anti-c-kit (APC, clone 2B8, Thermo Fisher Scientific 17-1171-81), anti-F4/80 (PE, clone BM8, Thermo Fisher Scientific MF48004), anti-Flt3 (PerCP-eFluor710, clone A2F10, Thermo Fisher Scientific 46-1351-80), anti-IgD (FITC, clone 11-26c.2a, BD Biosciences 562022), anti-IgM (APC, clone II/41, BD Biosciences 550676), anti-NK1.1 (PE, clone PK136, BD Biosciences 557391), anti-Sca-1 (PE-Cy7, clone D7, BD Biosciences 558162), Lineage cell detection cocktail (Biotin, Miltenyi Biotec 130-092-613), anti-H3K27me3 (EMD Millipore 07-449), anti-histone H3 (Cell Signaling Technology 9715), anti-rabbit IgG-HRP (Santa Cruz Biotechnology, Inc. sc-2004), anti-β-actin (clone AC-15, Sigma-Aldrich A5441), goat anti-mouse IgG-HRP (Santa Cruz Biotechnology, Inc. sc-2005), anti-VCAM-1 (Purified, clone 429, BD Biosciences 553330), anti-CD45.2 (PerCP-Cy5.5, clone 104, BD Biosciences 552950), anti-Bmi1 (Santa Cruz Biotechnology, Inc. sc-10745), anti-Cbx7 (Santa Cruz Biotechnology, Inc. sc-70232), anti-H2AK119ub (clone D27C4, Cell Signaling Technology 8240), anti-H3K9ac (Merck Millipore 06-942), anti-H3K4me3 (clone mAbcam12209, Abcam ab12209), anti-H3K27me3 (Merck Millipore 07-449), anti-Phc2 (Santa Cruz Biotechnology, Inc. sc-160664), anti-Ring1b (clone D22F2, Cell Signaling Technology 5694)
Validation	All antibodies were validated for the application and species used in this study by their manufacturers.

Eukaryotic cell lines

Policy information about cell lines

Cell line source(s)	OP9 (ATCC CRL-2749)
Authentication	None of the cell line used were authenticated.
Mycoplasma contamination	cell line was not tested for mycoplasma contamination.
Commonly misidentified lines (See ICLAC register)	N/A

Animals and other organisms

Policy information about studies involving animals; ARRIVE guidelines recommended for reporting animal research

Laboratory animals	C57BL/6 mice or gene targeted mice on a C57BL/6 background were used
Wild animals	not used
Field-collected samples	not used
Ethics oversight	All animals received proper care in accordance with the National Institutes of Health Guide for the Care and Use of Laboratory Animals. The study protocol was approved by the Institutional Animal Care and Use Committee of Korea University (protocol numbers: KUIACUC-20110104-3, KUIACUC-20141002-4 and KUIACUC-20160927-3).

Note that full information on the approval of the study protocol must also be provided in the manuscript.

Flow Cytometry

Plots

Confirm that:

☒ The axis labels state the marker and fluorochrome used (e.g. CD4-FITC).

☒ The axis scales are clearly visible. Include numbers along axes only for bottom left plot of group (a 'group' is an analysis of identical markers).

☒ All plots are contour plots with outliers or pseudocolor plots.

☒ A numerical value for number of cells or percentage (with statistics) is provided.

Methodology

Sample preparation	Single cell suspensions were prepared from the BM, PB, thymus, and spleen of each mouse. After removing red blood cells, cells were stained with MACS buffer at 4 °C in the presence of Fc Block (BD Biosciences 553141, dilution 1:100). After washing several times with PBS, stained cells were resuspended in PBS and analyzed by flow cytometry .
Instrument	Data was collected using FACSCalibur (BD Biosciences). Sorting was performed on a FACSAria Fusion Cell Sorter (BD Bioscience)
Software	Collection was performed using CellQuest software and analysis usig FlowJo software
Cell population abundance	Reanalysis of post-sort fractions<br>Lin-Sca-1+c-kit+ LSK cells>95%
Gating strategy	Supplementary figure 19 provide gating strategy for LSK cell sorting

☒ Tick this box to confirm that a figure exemplifying the gating strategy is provided in the Supplementary Information.
